# Tumor-Associated Macrophages (TAMs) Form an Interconnected Cellular Supportive Network in Anaplastic Thyroid Carcinoma

**DOI:** 10.1371/journal.pone.0022567

**Published:** 2011-07-21

**Authors:** Bernard Caillou, Monique Talbot, Urbain Weyemi, Catherine Pioche-Durieu, Abir Al Ghuzlan, Jean Michel Bidart, Salem Chouaib, Martin Schlumberger, Corinne Dupuy

**Affiliations:** 1 Department of Biopathology, Institut Gustave Roussy, Villejuif, France; 2 F.R.E. 2939, C.N.R.S., Villejuif, France; 3 U.M.R. 8126, C.N.R.S., Villejuif, France; 4 Unité 753, I.N.S.E.R.M., Villejuif, France; 5 Department of Nuclear Medicine and Endocrine Oncology, Institut Gustave Roussy, Villejuif, France; Florida International University, United States of America

## Abstract

**Background:**

A relationship between the increased density of tumor-associated macrophages (TAMs) and decreased survival was recently reported in thyroid cancer patients. Among these tumors, anaplastic thyroid cancer (ATC) is one of the most aggressive solid tumors in humans. TAMs (type M2) have been recognized as promoting tumor growth. The purpose of our study was to analyze with immunohistochemistry the presence of TAMs in a series of 27 ATC.

**Methodology/Principal Findings:**

Several macrophages markers such as NADPH oxidase complex NOX2-p22phox, CD163 and CD 68 were used. Immunostainings showed that TAMs represent more than 50% of nucleated cells in all ATCs. Moreover, these markers allowed the identification of elongated thin ramified cytoplasmic extensions, bestowing a “microglia-like” appearance on these cells which we termed “Ramified TAMs” (RTAMs). In contrast, cancer cells were totally negative. Cellular stroma was highly simplified since apart from cancer cells and blood vessels, RTAMs were the only other cellular component. RTAMs were evenly distributed and intermingled with cancer cells, and were in direct contact with other RTAMs via their ramifications. Moreover, RTAMs displayed strong immunostaining for connexin Cx43. Long chains of interconnected RTAMs arose from perivascular clusters and were dispersed within the tumor parenchyma. When expressed, the glucose transporter Glut1 was found in RTAMs and blood vessels, but rarely in cancer cells.

**Conclusion:**

ATCs display a very dense network of interconnected RTAMs in direct contact with intermingled cancer cells. To our knowledge this is the first time that such a network is described in a malignant tumor. This network was found in all our studied cases and appeared specific to ATC, since it was not found in differentiated thyroid cancers specimens. Taken together, these results suggest that RTAMs network is directly related to the aggressiveness of the disease via metabolic and trophic functions which remain to be determined.

## Introduction

Anaplastic thyroid carcinoma (ATC) represents less than 5% of all thyroid cancers and is one of the most aggressive malignancies in humans [1]–[3]. Despite multimodality therapeutic approaches, ATC still carries a dismal prognosis and new treatment, based on a better knowledge of the pathogenesis and progression of this cancer, are therefore required.

Anaplastic carcinoma arises from thyroid follicular cells and is characterized by atypical cells with large, bizarre nuclei with numerous atypical mitotic figures that resemble polymorphic mesenchymal sarcoma. There is no thyroid or epithelial differentiation. This explains why the macrophage component, termed “tumor-associated macrophages”, (TAMs), which is closely mixed with cancer cells, was only recently recognized [4], [5]. Among TAMs, M1 macrophages which display “classic” activation are separated from “alternative” M2 macrophages [6]–[10]. It is well established that the main function of M1 is phagocytosis in response to bacterial stimuli and/or Th1 cytokines while the main function of M2 is immunosuppression and trophic activity in response to Th2 cytokines (e.g. IL10, TGFbeta) [11]. In thyroid cancers, an increased density of TAMs was associated with decreased survival, reflecting their M2 nature [5].

In the present study, we report on a very dense and diffuse intra-tumor infiltration of ATC by TAMs. These TAMs organize themselves in an interconnected network in close contact with cancer cells and blood vessels.

## Material and Methods

### Clinical and Histological data

Twenty-seven patients who were treated for ATC at Institut Gustave-Roussy from 1998 to 2007 were identified. All these patients died within 1 year after diagnosis. Routine histological sections stained with hematoxylin and eosin were reviewed to confirm the diagnosis and corresponding paraffin-embedded tissue blocks were obtained for immunohistochemical studies. The protocol was approved by Institutional Review Board at the Institut Gustave-Roussy and all patients gave their written informed consent.

### Immunohistochemistry

Immunohistochemical analyses were performed on serial 5 microns thick sections prepared from selected paraffin-embedded tissue with primary antibodies directed against the following proteins: NOX2, P22phox, CD163, CD68, Cx43, Glut1, CD34, Alpha- Smooth Muscle Actin, CD3 and Ki67.

NOX2 is often referred to as the phagocyte NADPH oxidase and is still widely considered to have a limited, essentially phagocyte-specific tissue expression [12].
P22phox forms a heterodimer with NOX2 that is located at the cell surface membrane [12].
CD163 is expressed on most subpopulations of mature tissue macrophages [13] and is linked to anti-inflammatory macrophage functions [14].
CD68 has long been used as a specific macrophage marker [15], but is currently no longer considered a specific marker for macrophages but rather an antigen indicative of lysosomes [16].
Cx43 (connexin 43) is expressed by several types of cells including macrophages [17], [18] and is one of 21 members of the human connexin family. It is the most widely distributed gap junction isoform and enables the intercellular passage of numerous small molecules ranging from ions to much larger metabolites [18]–[20]. Subcellular Cx43 immunostaining correlates with protein synthesis in endoplasmic reticulum, the golgi apparatus, cytoplasmic transport in small vesicles and finally on cell surface membrane [21], [22].
Glut-1 is a glucose transporter that mediates cellular glucose uptake. It is inducible in monocytes and macrophages [23].
CD34 is a marker of vascular endothelial cells [24].
Alpha-Smooth Muscle Actin (Alpha-SMA) is present in “activated fibroblasts” or myofibroblasts [25]
CD3 recognizes lymphocyte T cells [26].
Ki67 nuclear expression is associated with cell proliferation [27].

The origin, characteristics and conditions of use of these antibodies are summarized in table 1.

**Table 1:** Immunohistochemistry: Antibodies, origins and procedures.

Antibody	Type	Origin	Dilution	Incubation time (mn)	Detection
**CD 163**	**monoclonal mouse**	**NOVOCASTRA Réf-NCL 163**	**1/100**	**60**	ENV MOUSE DAKO K4007
**NOX2 GP91-phox**	**monoclonal mouse**	**Marck Queen USA**	**1/300**	**60**	ENV MOUSE DAKO K4007
**P22-phox**	**polyclonal rabbit**	**SANTA CRUZ Ref.sc-20781**	**1/250**	**60**	ENV RABBIT DAKO K4011
**CX43**	**monoclonal rabbit**	**CELL SIGNALING Ref.3512**	**1/50**	**overnight**	ENV RABBIT DAKO K4011
**CD 34**	**monoclonal mouse**	**DAKO Ref.M7165**	**1/100**	**60**	ENV MOUSE DAKO K4007
**Alpha-SMA**	**monoclonal mouse**	**DAKO Ref.M0851**	**1/50**	**30**	ENV MOUSE DAKO K007
**CD68KP1**	**monoclonal mouse**	**DAKO Ref.N1577**	**1/400**	**60**	ENV MOUSE DAKO K4007
**CD3**	**monoclonal mouse**	**DAKO Ref.M7254**	**1/50**	**60**	ENV MOUSE DAKO K4007
**Ki 67**	**monoclonal mouse**	**DAK0 Ref.7240**	**1/40**	**30**	CSA II DAKO 1497

### Determination of percentage of TAMs among nucleated cells

**Figure 1:**
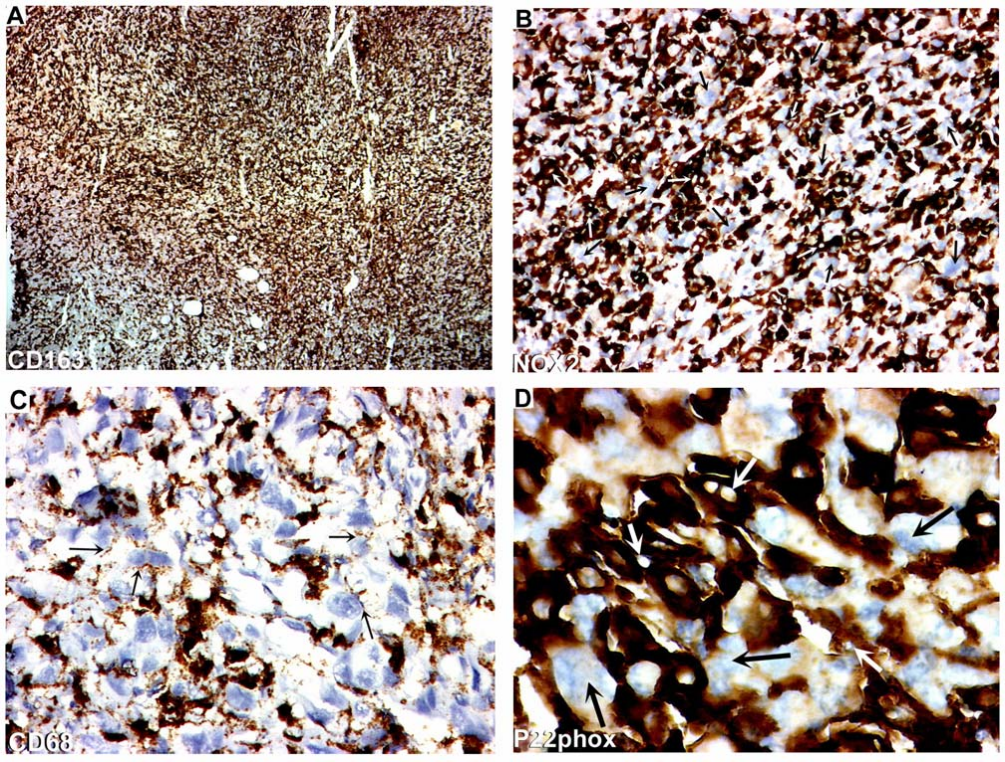
CD163, NOX2, CD68 and P22phox expressions in ATC. A) CD163. Original magnification: ×50. Presence of a very high density of positive RTAMs diffusely dispersed among negative cancer cells. B) NOX2. Original magnification: ×100. Positive RTAMs are deeply intermingled with negative cancer cells. Note inside RTAMs the presence of ovoid nuclei (white arrows) that are much smaller and more regular than those of cancer cells (black arrows). C) CD68. Original magnification: ×200. Note that immunostained cytoplasmic extensions of TAMs with granular features are in close contact with negative cancer cells (black arrows). D) P22phox. Original magnification: ×400. Note that the nuclei of cancer cells (black arrows) are five to ten-folds larger than RTAM nuclei (white arrows). All markers are strongly positive in RTAMs and negative in cancer cells and allow a clear distinction between these two cell types. The overall picture is the one of a chequered pattern with an even repartition of TAMs and cancer cells.

TAMs were counted by surface unit of histological section on digitalized photos with the same format and at the same original magnification (×100) with the help of ImageJ software (National Center for Biotechnology Information). For each case, four microscopic fields were digitalized; two with NOX2 immunostaining and two with CD163 immunostaining. The number and percentage of stained and unstained nucleated cells were calculated. To avoid any overestimation of the number of TAMs which could have been due to extended cytoplasmic ramifications, we counted only cells with a visible nucleus (Figs. 1–3, S1).

**Figure 2 pone-0022567-g002:**
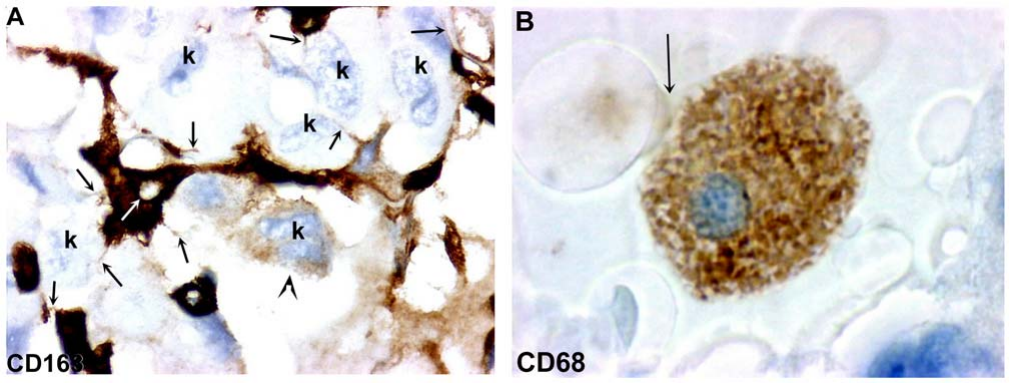
Morphologic comparison between RTAM (M2) and “classic” macrophage (M1). A) CD163. Original magnification: ×400. Positive RTAM (M2) with irregular cytoplasmic extensions exceeding 100 microns. Note very thin cytoplasmic processes (black arrows) with filipodia-like or nanotube-like appearances connecting RTAMs to other RTAMs or to cancer cells (K). Note the difference in the size of the RTAM nucleus (white arrow) and the size of cancer cell nuclei (K). Note that one cancer cell displays faint cytoplasmic staining (arrow head) suggesting the passage of antigenic material (CD163?) from the RTAM to the cancer cell. B) CD68. Original magnification:×400. “classic” M1 macrophage with a round shape and amoeboid appearance, without cytoplasmic and/or filipodia extensions. Presence of phagocytosis (arrow).

**Figure 3:**
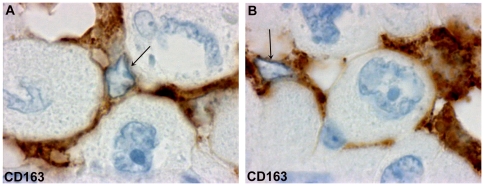
Topological relationship between RTAMs and cancer cells. CD163 immunostaining clearly underlines the close relations between cytoplasmic extensions of RTAMs and large cancer cells. Note that RTAMs nuclei (arrows) are much smaller than those of cancer cells. Original magnification: ×400.

### Determination of percentage of cancer cells and TAMs in the cellular cycle by double immunostaining with Ki67 and P22phox antibodies

See Text S1.

### Transmission electron microscopy

Three cases of ATC were obtained for electron microscopy study (See Text S1).

## Results

### Clinical data

Gender, age at the time of the diagnosis and survival of each patient are summarized in table 2.

**Table 2:** Percentage of RTAMs and proliferative rates of RTAMs and cancer cells.

N°patient	Age at diagnosis	Survival (months)	% RTAMs/Total	% RTAMs Ki67+/RTAMS	% Cancer cells Ki67+/cancer cells
1	79	4	56%	52%	50%
2	49	10	68%	41%	49%
3	73	0.2	21%	55%	57%
4	74	1	64%	38%	40%
5	85	0.5	57%	55%	58%
6	73	8	53%	22%	25%
7	82	2	57%	55%	52%
8	69	11	50%	29%	21%
9	55	2	58%	55%	58%
10	81	3	45%	43%	48%
11	82	1	52%	58%	51%
12	60	6	63%	49%	45%
13	93	3	62%	55%	58%
14	65	9	61%	50%	46%
15	74	1	60%	35%	45%
16	51	1	56%	37%	44%
17	72	0.3	66%	46%	49%
18	68	3	69%	23%	34%
19	59	8	52%	53%	58%
20	93	2	57%	40%	42%
21	61	3	55%	33%	27%
22	74	2	58%	44%	39%
23	84	4	68%	51%	48%
24	58	2	65%	53%	39%
25	70	11	50%	30%	22%
26	79	1	56%	53%	56%
27	56	4	65%	51%	49%

### NOX2, CD163 and CD68 immunostainings

TAMs displayed strong staining for NADPH oxidase NOX2, CD163 and CD 68 which clearly contrasted with cancer cells that were entirely negative for each of these markers. All ATC cases displayed a high density of TAMs that were diffusely dispersed within the tumor (Fig. 1). Furthermore TAMs and cancer cells could also be distinguished on morphological grounds. TAMs nuclei were small, regular and ovoid, whereas cancer cell nuclei were 5 to 10-fold larger, irregular and dystrophic (Figs. 1, 2A and 3). At the subcellular level, the strong staining obtained with all these markers, allowed us to study their cytoplasmic extensions. Three types of subcellular stainings were observed: a/ diffuse cytoplasmic staining ; b/ granular staining with small vesicles located in the cell body and/or in the cytoplasmic ramifications; c/ plasma membrane staining (Figs. 1,3 and S2). All these three types of staining were found in all the tumors according to the observed area.

### P22phox immunostaining

p22phox is required for the formation of a functionally active NADPH oxidase. Strong p22phox staining, similar to NOX2 staining, was found in all ATCs (Fig. 1D). However, in some areas of few cases, there was strong NOX2 positivity contrasting with weak p22phox staining. All cancer cells were negative for p22phox.

### Number and percentage of TAMs

In the 27 ATC studied, the mean proportion of TAMs was 57% of the total number of cells with a visible nucleus (Table 2). In fact, this percentage is likely to be a wide underestimation of the real percentage of TAMs since TAM nuclei were 5 to 10-fold smaller than cancer cell nuclei and the probability of cutting these nuclei on a 2D histological section is 5 to 10 times smaller than that of cutting cancer cell nuclei (Fig. 3).

### Shape and subcellular NOX2, CD163 and CD68 staining of TAMs

**Figure 4:**
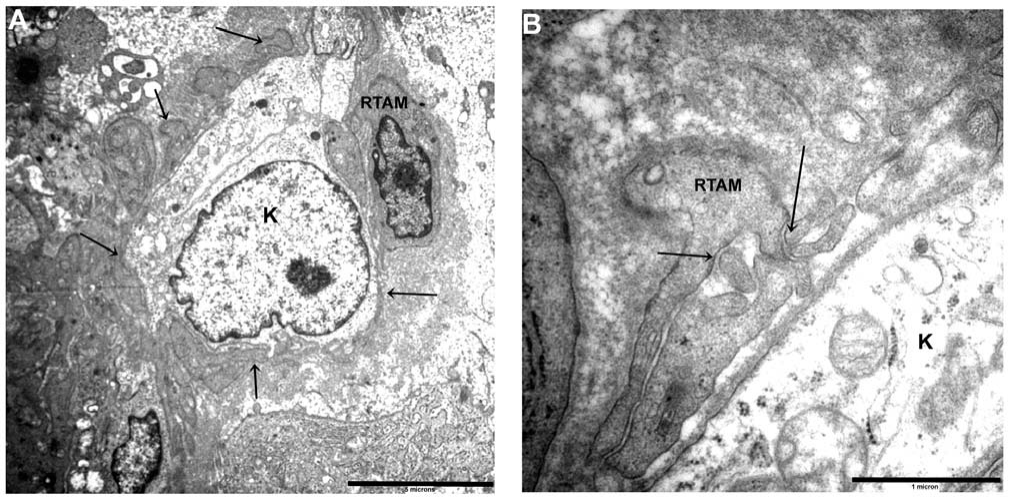
RTAMs and cancer cells at the ultrastructural level. A). Note the close relationship of RTAM and cancer cell. RTAM wraps its cytoplasmic processes (arrows) almost entirely around cancer cells (K). Original magnification: ×3000. B) Detail of RTAM cytoplasmic processes showing contacts with other processes (arrows) and their relations with cancer cell (K). Original magnification: ×12000.

Strong NOX2, CD163 and CD 68 stainings of TAMs allowed us to describe the following characteristics that were not visible with the usual hematoxylin and eosin staining: 1/ TAMs displayed very elongated cytoplasmic processes (up to 200 microns apart from the cell body); 2/ staining in these processes was often irregular with a moniliform pattern; 3/ these processes could divided in several more or less thinner branches; 4/ at their extremity, they often exhibited a small cytoplasmic ‘button shaped” enlargement; 5/ these extremities were in close contact with other TAMs, cancer cells and blood vessels; 6/ TAMs displayed no or very few intracytoplasmic phagocytic materials (Figs. 1, 2A, 3 and S2). Electron microscopy revealed cytoplasmic extensions of TAMs and close association with cancer cells (Fig. 4). We propose to call these TAMs “Ramified TAMs” (RTAMs). These features are in contrast to those of “classic” macrophages which present a more or less regular amoeboid round shape without major cytoplasmic extensions (Fig. 2B). Moreover this type of macrophages often presents intracellular phagocytized material.

### RTAM network

On NOX2, P22phox, CD163 and CD68 stainings, most TAMs displayed direct contact with other TAMs, forming networks of connected cells which could be tracked at least partially on 2D histological sections. These cellular networks connected TAMs to each other, enfolding dispersed isolated cancer cells and often in direct contact with them (Figs. 1, 3 and 4).

### Connexin 43 (Cx43) immunostaining

**Figure 5:**
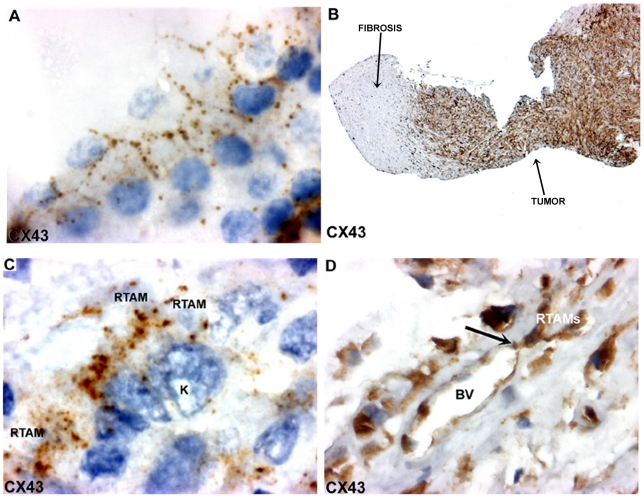
CX43 expression in normal thyroid tissue and in ATC. A) Normal thyroid tissue: characteristic CX43 positive punctuate gap junctions at the intercellular basolateral membranes of thyrocytes and absence of staining at the apical border. Note a faint diffuse or vesicular staining in the cytoplasm which could correspond to the synthesis and/or transport of the protein to the membrane. Original magnification: ×400. B) Strong CX43 immunostaining in ATC (on the right part of the photo). Non-tumor fibrotic tissue is seen on the left part. Original magnification: ×50. C). Characteristic CX43-positive punctuate junctions localized where the RTAMs and cancer cells (K) come into contact. Original magnification: ×1000. D) CX43 immunostaining of RTAMs and endothelial cells (BV: Blood Vessel). Note a CX43-positive “button” at the junction between the endothelial blood vessel and RTAM (black arrow). Original magnification: ×400.

**Figure 6:**
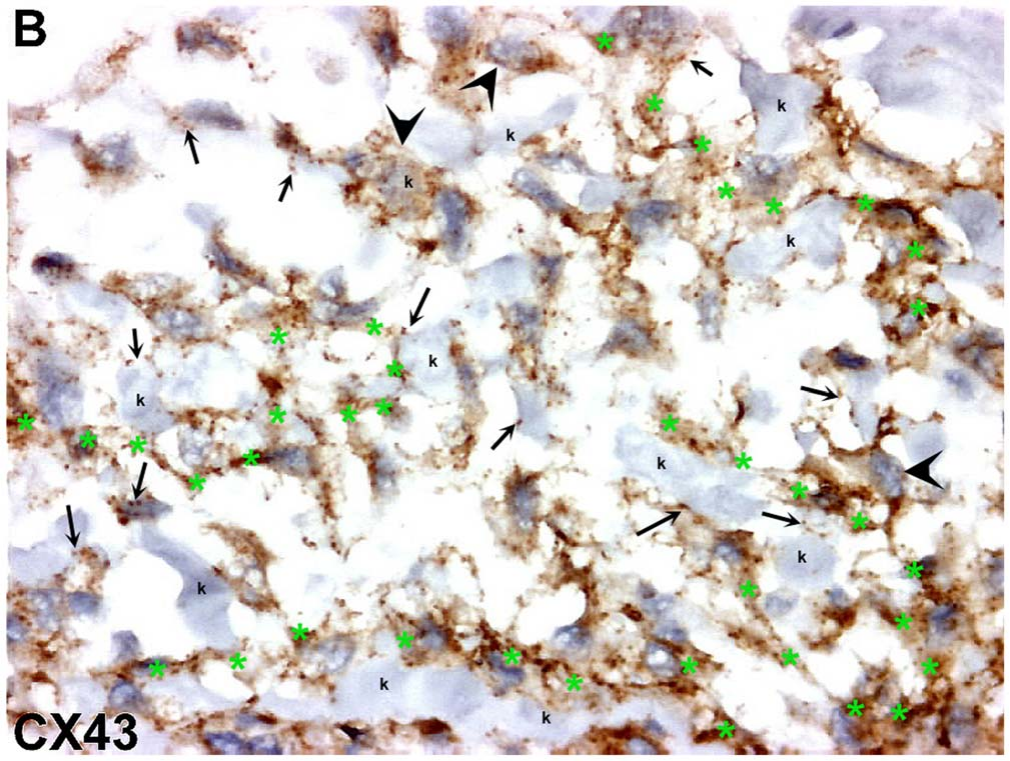
CX43 expression in ATC. CX43 immunostaining is mainly located in RTAMs. However, some cancer cells (k) display CX43 staining (black arrowheads). At the junction with cancer cells (small black arrows), presence of the characteristic punctuate pattern of gap junctions, as depicted in Fig. 4A. CX43-positive RTAMs interconnect to form an elongated network (green asterisks *). Original magnification: ×200.

**Figure 7:**
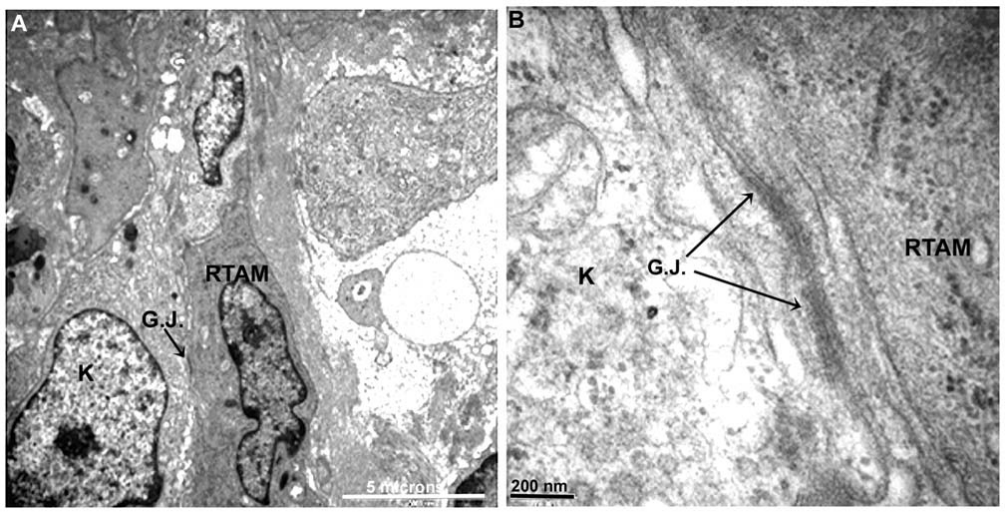
Demonstration of gap junctions at the ultrastructural level. A) Gap junctions between RTAM and cancer cell at low power (arrow). Original magnification: ×3000. B) The same gap junctions at high power (arrows). Original magnification: ×20 000.

In normal thyroid tissue, Cx43 staining was localized to characteristic punctuate structures at sites of cell-cell apposition (Fig. 5A). Cx43 positive staining was observed in all ATCs, in the majority of RTAMs and blood vessels and in a lower percentage of cancer cells (Figs. 5B, 5C, 5D and Fig. 6). Unlike in normal thyroid tissue, the staining was localized in the cytoplasm, the golgi area, small vesicles and/or characteristic intercellular punctuate structures suggesting a synthesis of CX43 and a more rapid turnover of the protein in ATC than in normal differentiated thyroid tissue [21]. Long chains of connected Cx43-positive RTAMs could be recognized and tracked within the tumor (Fig. 6). Moreover, it was possible to detect the Cx43 connection between positive blood vessels and RTAMs (Fig. 5D). At electron microscopy intercellular junctions consistent with gap junctions were found between RTAMs and between RTAMs and cancer cells (Fig. 7).

### Blood vessels

**Figure 8:**
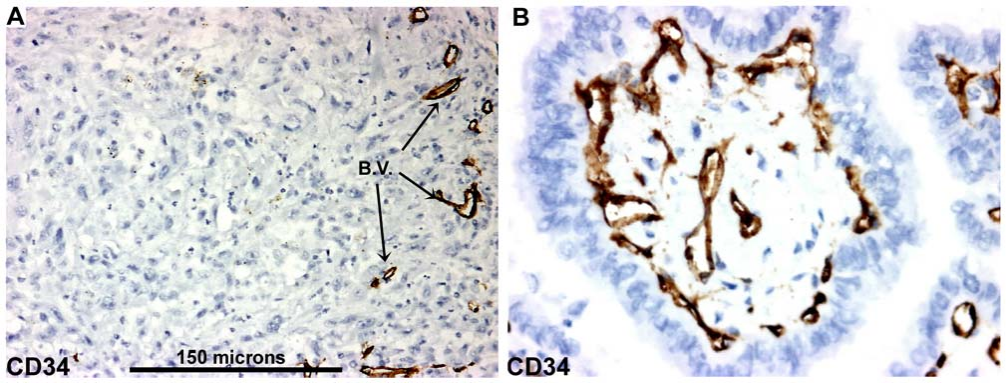
CD34 blood vessel immunostaining. A) Blood vessels display heterogeneous and unorganized distribution in ATC. Cancer cells could be located at a distance exceeding 150 microns from blood vessels (scale bar: 150 microns). Original magnification: ×100. B) CD34 immunostaining in a well-differentiated papillary thyroid carcinoma. Original magnification: ×200. Stained blood vessels follow the epithelial architecture and appear in close contact with cancer cells.

**Figure 9:**
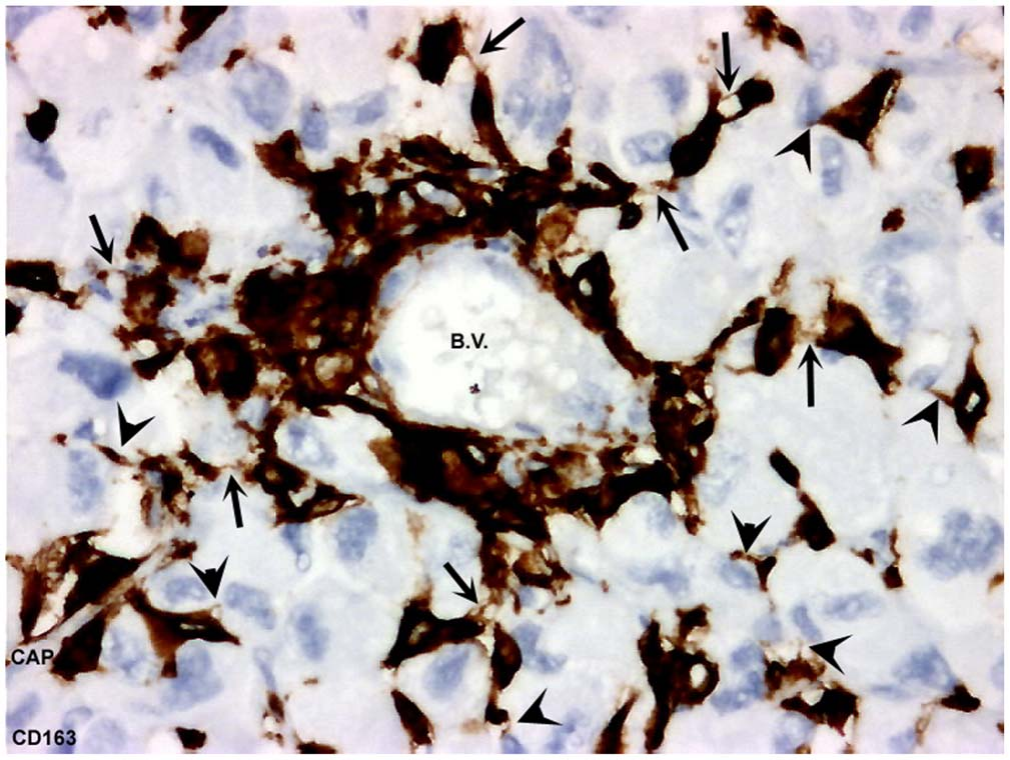
Cluster of RTAMs surrounding blood vessel. Original magnification: ×200. Cluster of CD163-positive RTAMs closely surround large blood vessel (B.V.) and small capillary (cap) and “stick together”. From this cluster, interconnected chains of RTAMs extend to within the tumor. Connections between RTAMs and cancer cells can be thick or very thin. (Long arrows show thin connections between RTAMS. Arrowheads show thin connections between RTAMs and cancer cells).

**Figure 10:**
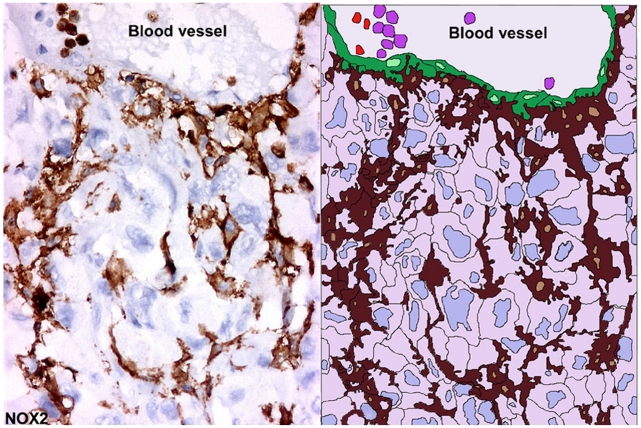
Extensions of interconnected RTAMs from blood vessel to avascular tumor areas. On the left, in case n°3 in which the number of RTAMs was relatively low and this allowed us to distinguish RTAMs along the blood vessel (B.V.) and the contiguous chain extension of RTAMs from the blood vessel to within the tumor cells. Original magnification: ×200. On the right, a drawing underlining the structure and the relationships between the different components was obtained by copying the photo in transparent digital layer with Adobe Photoshop and adding false colors; RTAMs in brown, cancer cells clear pink and blue; endothelial cells in green, neutrophils and/or monocytes in vessel lumen in violet and lymphocytes in red.

In well differentiated thyroid carcinoma immunostaining of blood vessels by CD34 displayed vasculature in close association with epithelial cancer cells. Almost each cancer cell is in direct contact with a blood vessel (Fig. 8B). In contrast, in ATC, vasculature was much more disorganized and anaplastic cancer cells could be localized in avascular areas at a distance of more than 150 microns from blood vessels (Fig. 8A). RTAMs were often located in clusters around blood vessels separating these vessels from cancer cells (Fig. 9), and long connected chains of RTAMs were infiltrating the avascular tumor tissue (Fig. 10).

### GLUT1 immunostaining

**Figure 11:**
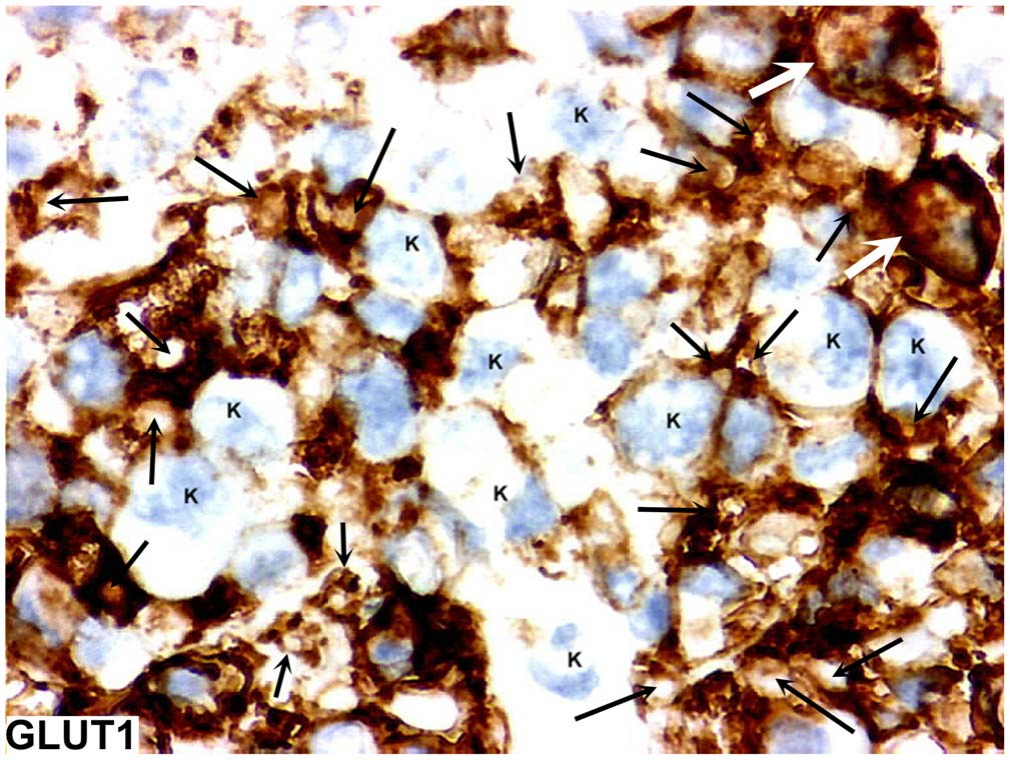
GLUT1 expression in RTAMs. Original magnification: ×200. GLUT1 positivity is more or less restricted to RTAMs (black arrows). Some rare cancer cells (k) are positive for GLUT1 (white arrows).

GLUT1 was positive in red blood cells and vessels. In some cases and in some areas, RTAMs were positive for GLUT1 whereas the great majority of cancer cells were negative for GLUT1 (Fig. 11).

### Single Ki67 immunostaining

In the majority of tumors, the proportion of Ki67-positive nuclei was around 50% (Table 2).

### Double Ki67-p22phox immunostaining

**Figure 12:**
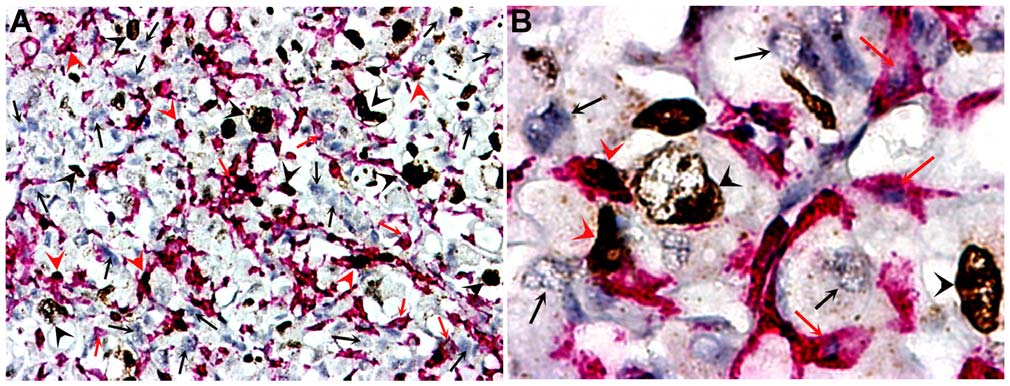
Double Ki67/P22 immunostainings. A) Original magnification: ×100; B) Original magnification: ×400. Ki67+ was revealed in brown-black color in the cell nuclei and P22phox in red in cytoplasm. Double immunostaining allowed distinguishing on the same section RTAMS Ki67+P22+ cells (red arrow-heads); RTAMs Ki67−P22+ cells (small red arrows); cancer cells Ki67+P22− (black arrow-heads) and Ki67−P22− (small black arrows).

This staining allowed recognizing four types of cells on the same section: Ki67− P22phox− and Ki67+ P22phox− cancer cells; Ki67− P22phox+ and Ki67+ and P22phox+ TAMs (Figs. 12 and S3). Each type of cells was counted with the help of the ImageJ software in the same way as for determining the whole percentage of TAMs (see Table 2). It appears that the mean proliferative rate of RTAMs was estimated to be 57% and the mean proliferative rate of cancer cells was estimated to be 45%.

### Alpha-SMA immunostaining

The staining was restricted to pericytes around blood vessels. All the other cells were negative. (Data not shown).

### CD3 immunostaining

This staining demonstrated an absence or very rare presence of CD3+ T lymphocytes (less than 0, 01%) (Data not shown).

### ^18^F-FDG PET/CT

All the patients suffering of ATC and studied at the institute Gustave-Roussy showed high to very intense FDG uptake. [28](Fig S4).

## Discussion

Nowadays, TAMs are classified in several subcategories according to different phenotypes that are dependent upon the type of tumors and microenvironments [29], [30]. In this study, we used NOX2, P22phox, CD163, CD68 and CD34 immunostainings to reveal and to characterize TAMs in ATC. With the use of these well recognized markers, it appeared that ATC tissue harbored only three types of cells: NOX2+ P22phox+ CD163+ CD68+ CD34− macrophages; NOX2− P22phox− CD163− CD68− CD34− cancer cells and NOX2− CD163− CD68− CD34+ blood vessel endothelium. Other cell types, such as lymphoid cells, fibroblasts and/or myofibroblasts were absent or very rare. Moreover ATC cancer cells were totally negative for all of these markers. This study confirms the presence of a high number of TAMs in most ATC that represent the majority of intra-tumor nucleated cells. Furthermore immunohistochemistry allowed us to accurately describe the morphology and the organization of TAMs.

Then we studied the ramified morphology of these TAMs and CX43 expression: TAMs in ATC displayed long and thin cytoplasmic processes which could extend up to 150 microns from the cell body. More often, these elongated processes were irregular and moniliform containing small stained vesicles, and were usually divided into several branches conferring a “microglia-like” appearance (Figs. 2A,3,4,S2). We propose to call them “Ramified TAMs” or “RTAMs”. RTAMs were in direct contact with other RTAMs, with cancer cells and with blood vessel endothelium. We suggest that these ramifications correspond to specific functions of type M2 TAMs and contrast to the absence of ramifications and the amoeboid shape of classic type M1 macrophages (Fig. 2B). Our hypothesis is that these cytoplasmic extensions of RTAMs may permit “cross talk” and molecular transfers between RTAMs and cancer cells. This “symbiotic” feature has been considered as a requirement for tumor aggressiveness [31].

Although differently expressed, CX43 was observed in the three cell types and at their interfaces. The great majority of RTAMs displayed CX43 positivity in the cytosol, in small vesicles either located in the cell body or along cytoplasmic processes and at appositional membrane areas with characteristic “punctuate” gap junction appearances, corresponding to the different phases of synthesis and processing of the protein [21].

As CX43 allows the intercellular passage of numerous small molecules ranging from ions to larger metabolites, these findings support our hypothesis that gap junctions between TAMs allow an efficient metabolic support by coordinating intercellular signaling and connections to cancer and endothelial cells.

**Figure 13:**
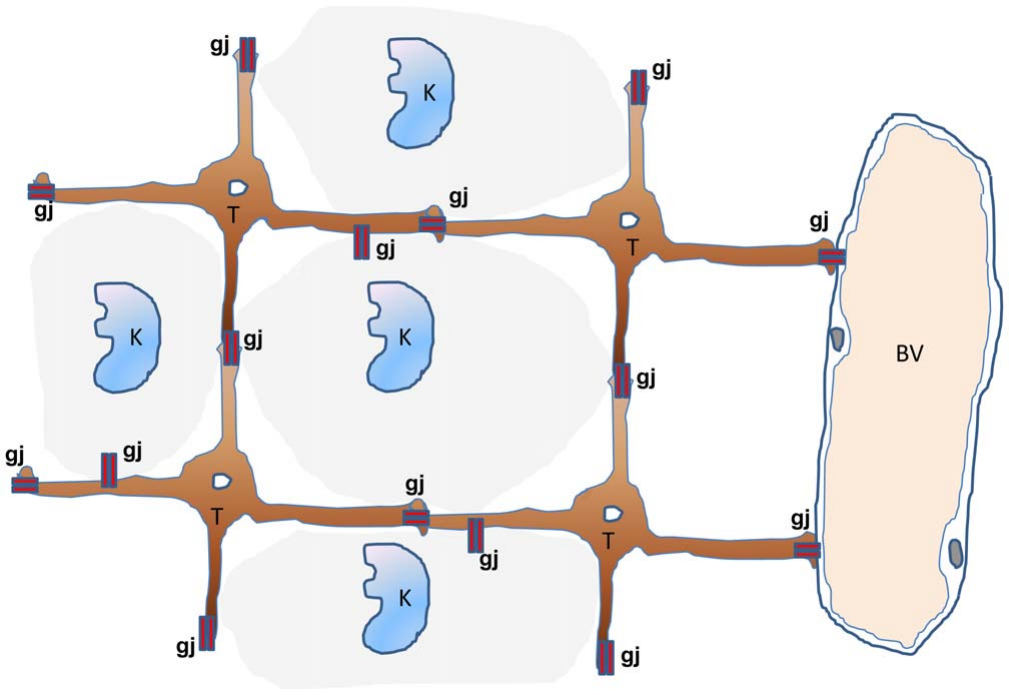
Schematic representation of TAMs network. TAMs are linked together via CX43 connexins (gap junctions) which could provide the network with a mechanism of intercellular communication between TAMs, their neighboring cancer cells and blood vessels.

We have schematically represented TAMs network on figure 13. A biological network structure is much more robust and resistant to diverse types of aggression or dysfunction than a non-interconnected structure [32]–[34]. This could give to ATC a definitive advantage on the neighboring non-tumor tissues

The vasculature could be considered the basis for the unique physiology of solid tumors in comparison with normal tissue [35]–[37]. In well-differentiated thyroid cancer, tumor cells appear in close contact with blood vessels like thyrocytes in normal thyroid tissue. In contrast, many cancer cells in ATC were located at a distance of more than 150 microns from blood vessels (Fig. 8A). This distance is considered to be the maximum distance of oxygen diffusion in tissues from blood vessels [38], [39]. However, these cancer cells, in direct contact with RTAMs did not show any sign of necrosis or apoptosis. Moreover, RTAMs were strikingly clustered around blood vessels and from these cell clusters, long chains of connected RTAMs extended to avascular areas, as attempting to establish contact with distant cancer cells (Fig. 9 and 10).

Several mechanisms other than tumor vasculature can provide the tumor with oxygen and nutrients [40]. Monocytes/macrophages may have the capacity to mimick blood vessels [41], in the *in vitro* formation of cord-like structures or branched cell columns in matrigel under angiogenic conditions [42]–[44] and in the *in vivo* transformation of monocytes into blood vessels [45], [46]. Besides morphological features, lineage markers were found to be co-expressed by both endothelial cells and monocytes [42] [47], [48]. However, in our study RTAMs and blood vessels were constantly clearly distinct since RTAMs were CD163+, CD34− and blood vessels CD163−, CD34+. Glod et al. underlined [46] that “the continued expression of myeloid proteins implies that the cells do not trans-differentiate into mature endothelial cells, but rather take on specific endothelial characteristics while remaining inherently monocytic cells.” Therefore, it is tempting to speculate that the interconnected RTAM network plays a vessel-like role in supplying nutrients from the blood to cancer cells.

Staining of Ki67 showed that the majority of cells were in a proliferative state and the double immunostaining for Ki67 and P22phox showed that the proliferation rates of RTAMs and cancer cells were roughly similar (Table 2). Thus these rapidly growing tumors display apparently the same RTAMs/cancer cells ratio during their evolution in accordance with the “symbiotic” hypothesis.

GLUT1 protein has been recognized as the main isoform of glucose transporters in malignant tumors [49]. Stimulation of glycolysis is an activation signal of macrophages [50]. In ATC, the fact that the majority of cancer cells were negative for GLUT1 and that only blood vessels and a subset of RTAMs were positive, supports the notion that glucose uptake from the blood takes place mainly in RTAMs and not or to a far lesser degree in cancer cells. Indeed GLUT1 has also been recognized as the main transporter for the glucose analogue 2-[18F]-fluoro-2-deoxy-D-glucose (FDG) in positron-emission tomography (PET) [49], [51] and ATC displays an intense uptake of ^18^FDG [28], [52] (Fig S4). Furthermore Kubota et al. [53] showed that ^18^FDG uptake was 2–4 times higher in tumor-associated macrophages tissues than in tumor cells.

These data suggest that the high uptake of FDG in ATC could be mostly related to the high number of RTAMs and that RTAMs network is related to glucose metabolism in the tumor.

TAMs displayed strong staining for NOX2 and its partner p22phox. NOX2 is the catalytic subunit of NADPH oxidase which produces reactive oxygen species (O_2_^−^ and H_2_0_2_) in activated macrophages in order to kill bacteria. Thus, this pro-inflammatory activity appears to be related more to “classic” M1 macrophages than to that of anti-inflammatory M2 macrophages. However, recent studies have revealed other functions of the NADPH oxidase systems [12]. In this context, macrophages in rats could highly potentiate the invasive capacity of hepatoma tumor cells, both *in vitro* and *in vivo* [54], [55] and this was inhibited by superoxide dismutase and catalase, This indicates a role for superoxide (O_2_-) and H_2_O_2_, both of which are generated by NADPH oxydase. Moreover, these studies argued in favor of direct contact between macrophages and tumor cells. Furthermore, the immunosuppressive properties of NOX2 and H_2_O_2_ have been demonstrated [56], [57] and reactive oxygen species (ROS) generated by NADPH oxidase are necessary for invadopodia and podosome formation which facilitates invasive behavior [58]–[60]. ROS can also modulate cell proliferation and the continuous generation of H2O2 is required for these mitogenic effects [61].

In ATC, cancer cells were negative for both NOX2 and p22phox. p22phox is also the functional partner of other NOX isoforms such as NOX1, NOX3 and NOX4 [12], and its lack of expression indicates that ATC cancer cells do not express these NADPH oxidase systems and consequently do not produce superoxide and H_2_O_2_ on their own. ROS within cells act as secondary messengers in intracellular signaling cascades which induce and maintain the survival and oncogenic phenotype of cancer cells [62], [63]. Numerous types of cancer cells display innate H_2_O_2_ overproduction, and this has been correlated with increased malignant potential [56]–[58] [64], [65]. Given the absence of the H_2_O_2_ generator in ATC cancer cells and the symbiotic function of RTAMs, it may be hypothesized that H_2_O_2_ produced by RTAMs could fuel cancer cells through interconnecting mechanisms such as Cx43. Moreover, beyond its role as a gap junction on the plasma membrane, Cx43 induces resistance to H_2_O_2_-mediated apoptosis, thus conferring an advantage for tumor growth [66].

Although essentially descriptive, our study demonstrates the presence in ATC of a dense and diffuse interconnected TAMs network. To our knowledge this is the first time that such a network is described in a malignant tumor. This network found in all of our studied ATC cases appears related to the anaplastic nature of the proliferation since it was not present in more differentiated thyroid carcinomas and implies that the cancer cells were isolated by TAMs ramifications. We proposed some hypotheses concerning its possible functions given the aggressiveness of the disease and its need in a great amount of energy. Further studies using experimental models are now warranted to investigate the role of such interconnected macrophage network. Finally, TAMs were recently manipulated *in vivo* and *in situ* with pharmacological agents or cytokines to induce cytotoxic activity against cancer cells [67]–[69]. Given its dismal prognosis, ATC appears to be a candidate for such therapeutic approaches.

## Supporting Information

Text S1 **Supplemental material and methods.**
(DOC)

Figure S1 **Count of nuclei RTAMs and nuclei cancer cells.** On the left original NOX2 immunostaining. Original magnification ×100. On the right nuclei are stained in false colors. The count is made with the help of Imagej software. TAMs nuclei are type 1 and are colored in green and cancer cells nuclei are type 2 and are colored in blue. For this peculiar field the number of TAMs nuclei were 162 and the number of cancer nuclei 152 that to say a percentage of 52% of TAMs on total nuclei population.
(TIF)

Figure S2 **NOX2 (A) and CD163 (B) immunostainings in TAMs.** Strong intracytoplasmic staining for NOX2 and CD163. Note the granular appearance in cytoplasmic extensions (arrows). Magnification in A and B: ×1000.
(TIF)

Figure S3 **Double immunostaining Ki67/P22.** Ki67 is stained in brown/black in the nucleus and P22 is stained in red in the cytoplasm. The different types of cells according to their staining were counted with the help of ImageJ software: Type1 corresponds to TAMs Ki67− P22+; type2 to TAMs Ki67+, P22+; type3 to cancer cells Ki67− P22− and type4 to cancer cells Ki67+, P22−.
(TIF)

Figure S4 **^18^F-FDG PET/CT in a patient suffering of anaplastic thyroid carcinoma.** Note an intense uptake of the ^18^F-FDG by the thyroid tumor.
(TIF)
