# Supplementary material for: Tumor-Associated Macrophages (TAMs) Form an Interconnected Cellular Supportive Network in Anaplastic Thyroid Carcinoma
Source: PLoS One. 2011 Jul 21;6(7):e22567. doi: 10.1371/journal.pone.0022567 (PMC3141071; doi:10.1371/journal.pone.0022567)
Supplement: Text S1 — Supplemental material and methods. (DOC) [file pone.0022567.s001.doc]

**S1 Materials and methods:**

**Double immunostaining:**

5-microns sections were deparaffinized by serial passages in xylene and

in alcohol. Endogenous peroxidase activity was quenched by

incubation in 0.03% of hydrogen peroxide and in 0.1 M Tris-

HCl buffer 1X (pH 7.6) for 5 minutes. Subsequently, microwave/

pressure cooker pretreatment (three cycles of 5minutes each) was performed in 1 mM ethylenediaminetetraacetic acid (EDTA) buffer (pH 8).

Slides were first incubated with the Ki67 antibody (Mib1-Dako ref: M7240) at a 1:40 dilution for 30 minutes

They were washed three times in Tris-HCl 1X buffer for 5 minutes

each time and incubated with a peroxidase conjugated CSA II kit (Dako- ref:1497) for 15 minutes. ). After three additional washes, peroxidase was revealed in diaminobenzidine tetrahydrochloride (Polysciences

Inc, Warrington, PA) with 0.1% hydrogen peroxide,

in Tris buffer 0.01 M (pH 7.2). Ki67 immunostaining appears dark brown and is localized in cell nuclei.

Then, after three washes, they were incubated with the anti- p22 phox antibody (F1195-Santa Cruz- ref: sc20781) for x minutes. Slides were incubated an anti-rabbit phosphatase alkaline Powervision kit (Dako-ref:PU 6133). Phosphatase alkaline was revealed with permanenr red (Dako-ref: K0640). P22phox immunostaining appears red and is localized in cytoplasm.

Nuclei were counterstained with Meyer s’ Hematoxylin.

**Transmission Electron Microscopy:**

Small pieces (no more than 1mm^3^) of 3 cases of fresh AIT tumor tissue were fixed in 2.5% glutaraldéhyde and 0.1 M phosphate buffer (pH 7.2), immersed in 1% OsO_4_ for 1 h at 4 °C,

dehydrated by graded concentrations of ethanol, and embedded in Epoxy's resin according to standart procedures. Ultrathin sections (90 nm) were generated and double- stained with uranyl acetate and lead citrate and examine under a carl Zeiss EM 109 T transmission electron microscope.
