# Supplementary figures and images for: Tumor-Associated Macrophages (TAMs) Form an Interconnected Cellular Supportive Network in Anaplastic Thyroid Carcinoma
Source: PLoS One. 2011 Jul 21;6(7):e22567. doi: 10.1371/journal.pone.0022567 (PMC3141071; doi:10.1371/journal.pone.0022567)

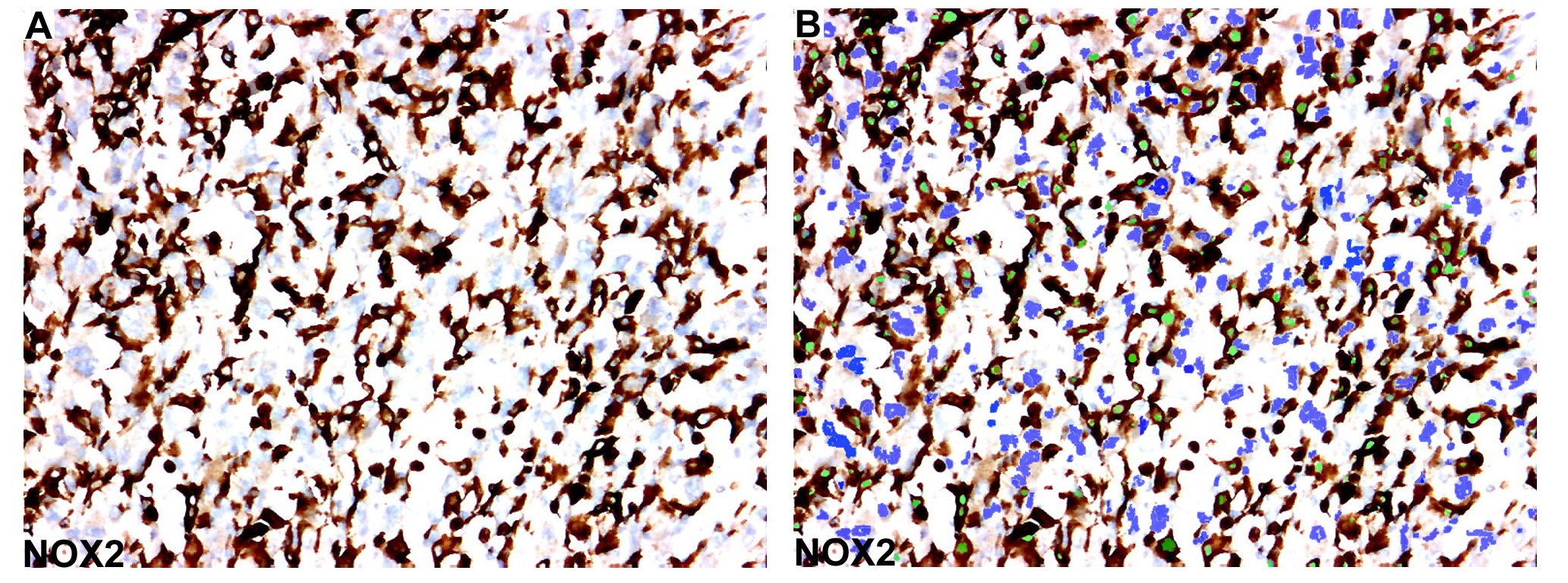

Supplement: Figure S1 — Count of nuclei RTAMs and nuclei cancer cells. On the left original NOX2 immunostaining. Original magnification ×100. On the right nuclei are stained in false colors. The count is made with the help of Imagej software. TAMs nuclei are type 1 and are colored in green and cancer cells nuclei are type 2 and are colored in blue. For this peculiar field the number of TAMs nuclei were 162 and the number of cancer nuclei 152 that to say a percentage of 52% of TAMs on total nuclei population. (TIF) [file pone.0022567.s002.tif]

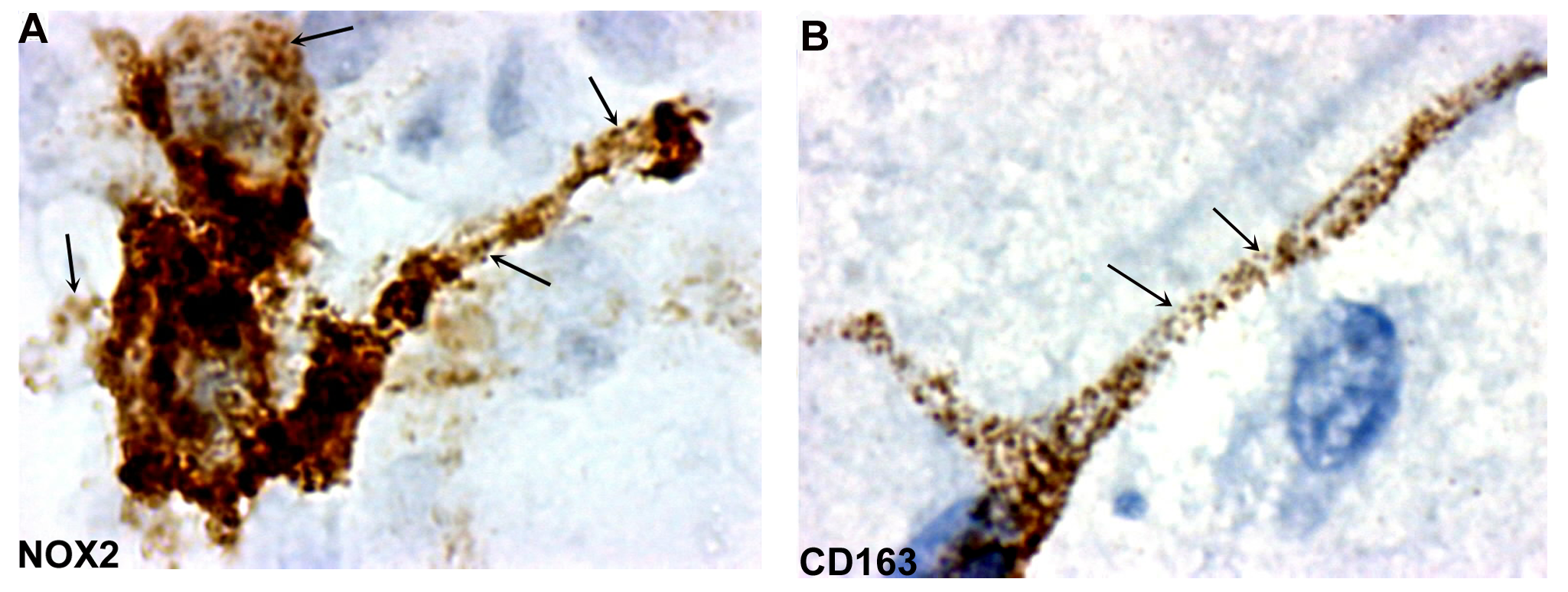

Supplement: Figure S2 — NOX2 (A) and CD163 (B) immunostainings in TAMs. Strong intracytoplasmic staining for NOX2 and CD163. Note the granular appearance in cytoplasmic extensions (arrows). Magnification in A and B: ×1000. (TIF) [file pone.0022567.s003.tif]

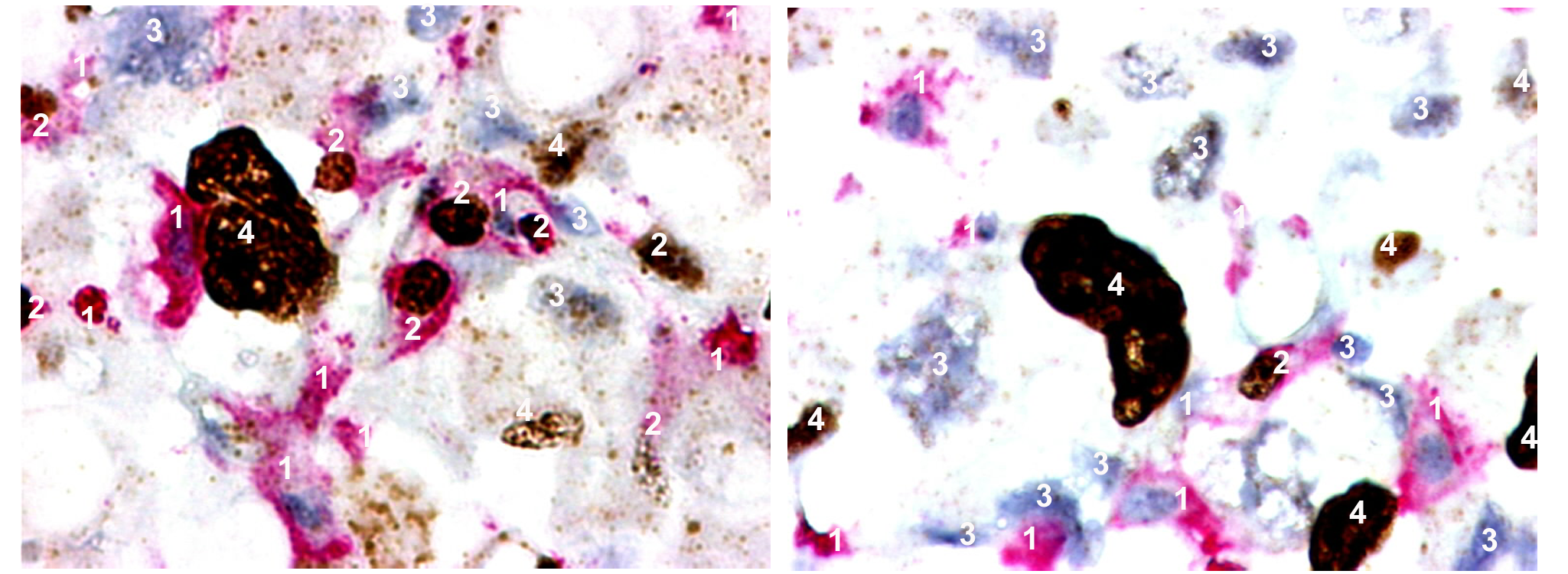

Supplement: Figure S3 — Double immunostaining Ki67/P22. Ki67 is stained in brown/black in the nucleus and P22 is stained in red in the cytoplasm. The different types of cells according to their staining were counted with the help of ImageJ software: Type1 corresponds to TAMs Ki67− P22+; type2 to TAMs Ki67+, P22+; type3 to cancer cells Ki67− P22− and type4 to cancer cells Ki67+, P22−. (TIF) [file pone.0022567.s004.tif]

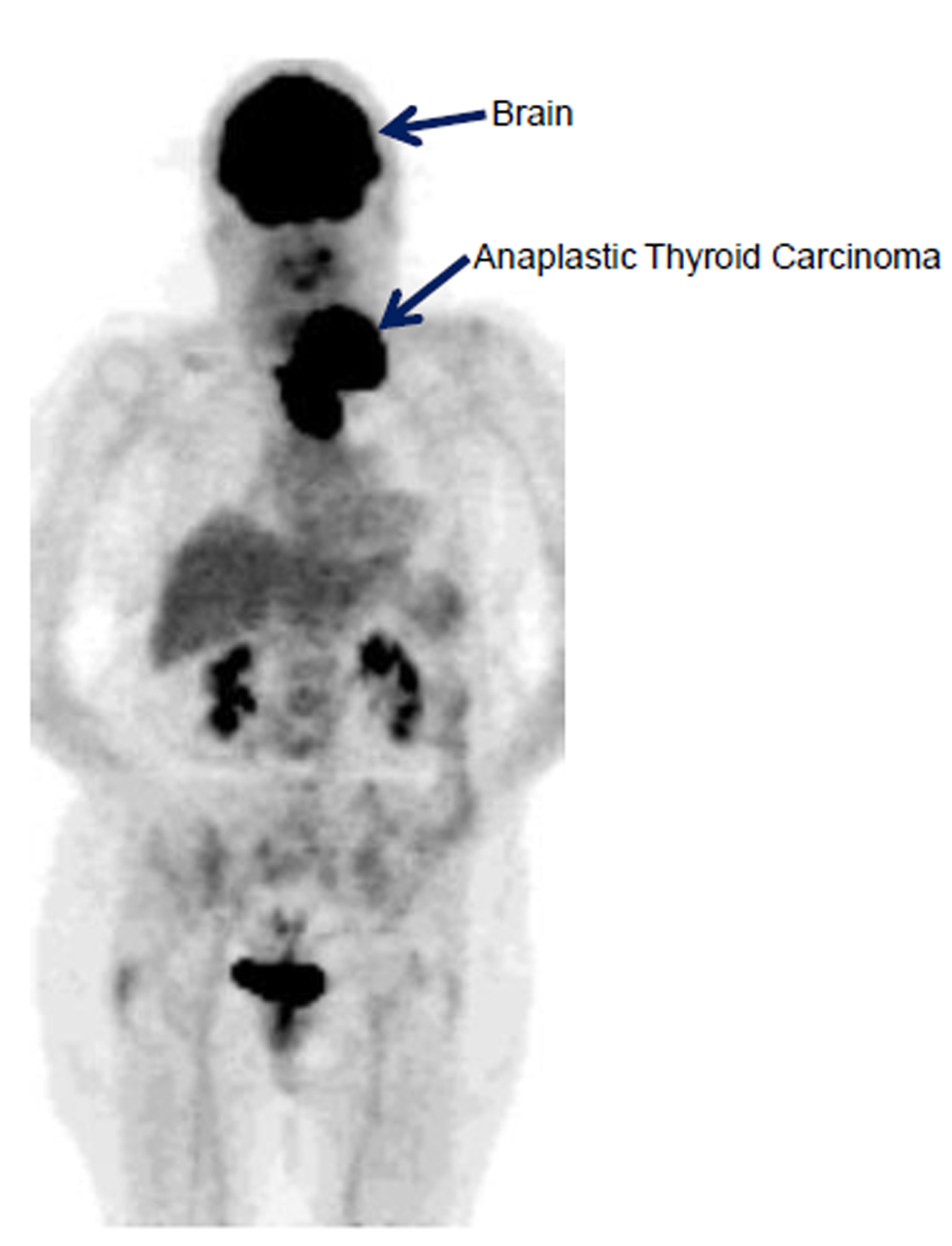

Supplement: Figure S4 — 18F-FDG PET/CT in a patient suffering of anaplastic thyroid carcinoma. Note an intense uptake of the 18F-FDG by the thyroid tumor. (TIF) [file pone.0022567.s005.tif]
